# A truncated HIV Tat demonstrates potent and specific latency reversal activity

**DOI:** 10.1128/aac.00417-23

**Published:** 2023-10-24

**Authors:** Ellen Van Gulck, Marion Pardons, Erik Nijs, Nick Verheyen, Koen Dockx, Christel Van Den Eynde, Emilie Battivelli, Jerel Vega, Eric Florence, Brigitte Autran, Nancie M. Archin, David M. Margolis, Christine Katlama, Chiraz Hamimi, Ilse Van Den Wyngaert, Filmon Eyassu, Linos Vandekerckhove, Daniel Boden

**Affiliations:** 1 Janssen Infectious Diseases, Janssen Research and Development, A Division of Janssen Pharmaceutica NV, Beerse, Belgium; 2 HIV Cure Research Center, Department of Internal Medicine and Pediatrics, Ghent University Hospital, Ghent University, Ghent, Belgium; 3 Janssen Infectious Diseases, A Division of Janssen Pharmaceutica NV, Brisbane, California, USA; 4 Arcturus Therapeutics, Science Center Drive, San Diego, California, USA; 5 Institute of Tropical Medicine, Antwerp, Belgium; 6 Faculty of Medicine Sorbonne-University, CIMI-Paris, UPMC/Inserm, Paris, France; 7 University of North Carolina School of Medicine and UNC, HIV Cure Center, Chapel Hill, North Carolina, USA; 8 Department Infectious Diseases, Hospital Pitié Salpetière, Sorbonne-University and IPLESP, Paris, France; 9 Discovery Sciences, Janssen Research and Development, A Division of Janssen Pharmaceutica NV, Beerse, Belgium; IrsiCaixa Institut de Recerca de la Sida, Badalona, Barcelona, Spain

**Keywords:** HIV, latency, Tat

## Abstract

A major barrier to HIV-1 cure is caused by the pool of latently infected CD4 T-cells that persist under combination antiretroviral therapy (cART). This latent reservoir is capable of producing replication-competent infectious viruses once prolonged suppressive cART is withdrawn. Inducing the reactivation of HIV-1 gene expression in T-cells harboring a latent provirus in people living with HIV-1 under cART may result in depletion of this latent reservoir due to cytopathic effects or immune clearance. Studies have investigated molecules that reactivate HIV-1 gene expression, but to date, no latency reversal agent has been identified to eliminate latently infected cells harboring replication-competent HIV in cART-treated individuals. Stochastic fluctuations in HIV-1 *tat* gene expression have been described and hypothesized to allow the progression into proviral latency. We hypothesized that exposing latently infected CD4+ T-cells to Tat would result in effective latency reversal. Our results indicate the capacity of a truncated Tat protein and mRNA to reactivate HIV-1 in latently infected T-cells *ex vivo* to a similar degree as the protein kinase C agonist: phorbol 12-myristate 13-acetate, without T-cell activation or any significant transcriptome perturbation.

## INTRODUCTION

The introduction of combination antiretroviral therapy [cART (1)] greatly increased life expectancy and improved the quality of life of people living with HIV, transforming HIV infection from a lethal to a chronic infection under cART (2). However, cART does not cure HIV infection due to the existence and persistence of a latent viral reservoir (3 – 5) that is established early after infection (6 – 8). The latent HIV reservoir is mainly formed as a small pool of long-lived memory CD4^+^ T-cells harboring an integrated latent provirus (5, 9). As the mean half-life of this latent reservoir is approximately 44 months in cART-treated individuals, complete viral elimination would require 70 years of continuous cART (9 – 11). HIV cure would result in significant health benefits by removing the substantial burden of daily treatment in addition to lifting the social stigma associated with HIV.

Several experimental approaches in HIV-infected individuals have been described to decrease the size of the HIV latent reservoir to potentially allow the discontinuation of cART without the risk of viral rebound: early cART administration (12); myeloablative therapy for malignancy followed by transplantation of cells lacking co-receptors for virus (13); vaccine immunotherapy (14, 15); block and lock strategy, which aims to permanently silence integrated provirus by using HIV latency-promoting agents (16); and the use of latency reversal agents (LRAs) in the presence of cART to induce latency reversal and clearance of the HIV (17 – 19).

Various classes of LRAs have been tested *ex vivo* individually or in combination in either immortalized CD4+ T-cell lines or primary CD4 cells from HIV-infected individuals. Examples of candidate LRAs include protein kinase C (PKC) agonists which activate multiple signaling pathways including the nuclear factor kB pathway (20 – 29), histone deacetylase inhibitors which induce HIV gene expression by facilitating a more transcription permissive chromatin environment (17, 19, 30 – 33), bromodomain inhibitors (34 – 38), toll-like receptor (TLR) agonists (39 – 42), AK strain transforming (AKT) agonists (43 – 46), second mitochondria-derived activator of caspases (SMAC mimetics) (47), and immunomodulators (32, 48 – 50). In initial clinical trials, these LRAs alone or in combination enhanced cell-associated HIV RNA levels, but none of these compounds led to a significant decrease in the size of the latent HIV reservoir in people living with HIV [reviewed in references (51, 52)]. As follow-up to shock and kill strategies, PKC agonists or immunomodulators were combined with other interventions such as therapeutic vaccination or broadly neutralizing antibodies. These therapies have shown promising results in non-human primates leading to the reduction of the viral reservoir or extended periods of aviremia (53 – 55). Recently, Kim et al. showed that a single administration of the PKC modulator SUW133 combined with allogenic human peripheral blood NK cells delayed viral rebound after treatment interruption in humanized mice infected with HIV (56). There remains a need to enhance our understanding of the biology of the latent reservoir as well as to develop novel LRAs that are potent, specific, and tolerable for repeated administration.

HIV transcription is driven by its native 5′-long terminal repeat (LTR) promoter whereby transcriptional activity is auto-induced by the HIV-1 transactivator of transcription (Tat) protein leading to a powerful positive feedback loop (57). The transactivation capacity of Tat was reported shortly after the discovery of HIV showing a 200- to 300-fold LTR stimulation of integrated proviral transcription (58 – 60). HIV Tat recruits the host positive transcription elongation factor b (P-TEFb) along with multiple additional transcription factors to a stem-loop RNA structure called transactivation response region (TAR) (61) which is located immediately downstream of the LTR transcription start site. The P-TEFb complex is composed of the cyclin T1 and CDK9 subunits whereby the latter promotes the phosphorylation of the RNA polymerase II (RNAPII) C-terminal domain and additional regulatory proteins (62 – 64), leading to the full activation of polymerase processivity and elongation of HIV transcripts.

Several *in vitro* studies have shown that different levels of Tat expression can define the fate of HIV-infected cells by either producing infectious particles or establishing latency (65). It was shown that the introduction of exogenous Tat was able to induce latency reversal both in a latently infected cell line model (66) as well as in memory CD4+ T-cells from cART-treated individuals *in vitro* (65, 67), independently of the chromatin environment surrounding the integration site (65). While intracellular expression of Tat prevents latency establishment, HIV infection with an attenuated Tat virus increases the frequency of latently infected cells (68). Stochastic fluctuations of Tat expression might act as a molecular switch between active transcription and latency (69 – 71).

The HIV-derived accessory protein Tat (86–101 aa) is translated from two different exons where exon 1 (1–72 aa) contains all the domains essential for transactivation (72). Here, we seek to reduce the size of Tat to the minimum protein domains that are critical for its reactivation capacity by deleting regions that upon non-specific cell receptor binding may activate off-target signaling cascades. We explored various deletion mutants of Tat as potential candidates for latency reversal, both in cell lines and in primary CD4+ T-cells from cART-treated HIV-1-infected individuals. If low level of Tat expression plays a role in the establishment of HIV latency, we hypothesized that providing sufficient Tat protein in *trans* would result in efficient HIV-1 reactivation. In this study, we identified a Tat deletion mutant (T66) to be as efficient as full-length Tat protein to induce latency reversal *in vitro* and *ex vivo*. Importantly, the mutant did not induce global T-cell activation in primary CD4+ T-cells, nor did it lead to any significant perturbation of the human T-cell transcriptome. Furthermore, we investigated delivery of T66 mRNA to CD4+ T-cells via lipid nanoparticles and observed significantly enhanced HIV reactivation when compared to the T66 protein. The enhanced activity of T66 mRNA delivered by nanoparticles is likely due to more efficient cytosolic delivery as opposed to T66 protein transduction which depends on endocytic uptake mechanisms and endosomal escape of the entrapped protein.

## RESULTS

### Identifying minimal HIV Tat activation domain

**Fig 1:**
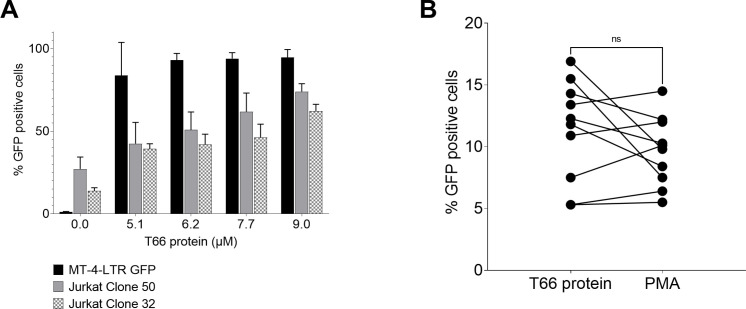
Identifying minimal Tat construct that retains maximal transactivation capacity. (**A**) HEK293 T-cells were co-transfected with different Tat expression plasmids (on x-axis), pLTR-Fluc and pEF-Rluc plasmids. LTR activity was assessed via the firefly luciferase read-out along with signal normalization by Renilla luciferase. Data are normalized to LTR activity of Tat-72 construct (exon 1) which contains all the domains essential for transactivation. Replicate number *n* ≥ 4. Mean, error bars indicate SEM. Dunnett’s multiple comparison test was performed to evaluate statistical significant difference compared to Tat-72 [****P < 0.0001; ***P < 0.0005, **P < 0.005, *P < 0.05, otherwise not significant (ns)]. (**B**) Production of HIV RNA from CD4+ T-cells: three cART-treated donors from cohort A (donor 1 until donor 3, indicated on the x-axis) were incubated for 24 hours with medium (black bar), Tat-66 (dark gray bar), Tat-60 (patterned bar), and Tat-86 (light gray bar) protein. HIV-1 reactivation following latency reversal was assessed by reverse transcription-quantitative real-time PCR. Mean ± SD (*n* = 20 replicates for donor 1, *n* = 16 for donor 2, and *n* = 8 replicates for donor 3) of relative HIV-1 gag RNA copies for each donor are plotted on the y-axis. For statistical analysis, non-parametric Kruskal-Wallis test was used: ***P < 0.0005, ****P < 0.0001, otherwise not significant (ns).

A deletion exercise was performed to delineate the essential Tat amino acid sequences that would result in the same HIV-LTR activation capacity as the 72 aa Tat exon 1 protein. The intention was that reducing the overall size of HIV Tat will (i) remove potential anti-HIV Tat antibody epitopes, (ii) improve cellular uptake, (iii) diminish off-target effects due to reduced non-specific binding, and (iv) facilitate chemical polypeptide synthesis. The latter was chosen over bacterial or yeast recombinant protein production to exclude any TLR ligand contamination in the Tat protein. Several Tat deletion mutants, ranging from 57 aa up to 86 aa, were introduced into plasmid DNA (pDNA) expression vectors. These constructs were used to co-transfect HEK293 cells along with pLTR-FLuc and pEF-RLuc reporter plasmids. Tat-86 and Tat-72 showed similar transactivation activity confirming the reported finding that exon-1 (72 aa) contains all elements essential for transactivation (73, 74). The Tat variant comprising only 66 amino acids was identified to have comparable transactivation activity as the full-length exon Tat-72 construct, whereas all smaller variants showed reduced reactivation activity (Fig. 1A). The reduced transactivation potency of shorter Tat mutants could be due to loss of transactivation activity or impaired protein stability. The reduced transactivation potency was further evaluated with a newly synthesized Tat-60 protein which contains the core and TAR binding domains essential for Tat-mediated LTR activation. The activation potential of Tat-60, Tat-66, and recombinant Tat-86 was tested in CD4+ T-cells isolated from cART-treated individuals (Fig. 1B). For induction in primary cells, we used a maximum concentration of the T66 protein that maintained high cell viability (4 µM; Fig. S1). The lower overall Tat-60-induced activation confirms the results of previous transfection data showing a reduced reactivation capacity of Tat-60 compared to Tat-66 and Tat-86. This work further explored the latency reversal potential of the Tat-66 variant in different *in vitro* and *ex vivo* latency reactivation assays.

### Strong and consistent T66 protein-mediated LTR activation of latent HIV in cell lines and primary CD4 T-cells

**Fig 2:**
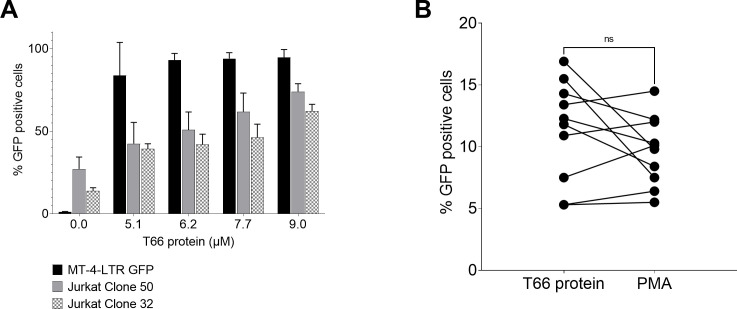
Evaluation of the capacity of the T66 protein to activate LTR in different engineered latently infected T-cell lines and CD4+ reporter cells. (**A**) Three different HIV-LTR GFP reporter T-cell lines: MT-4-LTR GFP (black bars), Jurkat Cl50 (gray bars), and Jurkat Cl32 (patterned bars) were incubated for 16 hours with different concentrations of T66 protein (x-axis). LTR activation in these cell lines resulted in GFP expression, which was measured by flowcytometry. Percentage of GFP-positive cells is plotted on the y-axis. The plot represents the mean of *n* = 4 ± SD for MT-4-LTR GFP and Jurkat CL32 and mean of *n* = 3 ± SD for Jurkat CL50. (**B**) Primary CD4+ T-cells from 10 different HIV-seronegative donors were isolated and co-infected with an HIV dual reporter virus and a lentivirus expressing pro-survival proteins. Following infection, latently infected cells were sorted and exposed for 24 hours to PMA (5 ng/mL) or T66 protein (4 µm). On the Y-axis, increase in percentage of GFP-positive cells compared to negative (PBS-treated cells) is plotted for both stimuli. For statistical analyses, non-parametric Wilcoxon tests were used. Statistically significant *P* values are represented on the graphs. *P < 0.05.

We first evaluated latency reversal following a dose response with the T66 protein (ranging from 5.1 to 9 µM) in three different HIV reporter cell lines: MT-4-LTR-GFP, Jurkat clone 50, and Jurkat clone 32 (Fig. 2A). The percentage of GFP-positive cells reached a plateau at a concentration of 6.2 µM in the MT-4 cell line with 93% of GFP-positive cells. In Jurkat clone 50 and 32, a dose response is seen up to 9 µM. To confirm these results in a more physiologically relevant model, a transient HIV reactivation assay was established in primary CD4+ T-cells. This assay is based on the co-infection of isolated CD4+ T-cells from HIV-seronegative donors with an HIV reporter construct and a virus expressing two antiapoptotic proteins (MCL1 and Bcl2). Following the establishment of quiescent proviral DNA and the subsequent overnight incubation of these CD4+ T-cells with phospate-buffered saline (PBS), T66 protein, or phorbol 12-myristate 13-acetate (PMA), substantial levels of reactivation were obtained with T66 and PMA in all donors tested, expressed as percent increase in GFP compared to PBS-treated control (Fig. 2B). While the LTR reactivation of T66-treated cells exceeded the LTR reactivation achieved by PMA stimulation in 5 out of 10 donor samples, T66 and PMA showed generally similar reactivation capacity (*P* = 0.07) in this assay system.

### Strong and consistent latency reversal activity in CD4 T-cells from cART-treated individuals

Next, the latency reversal capacity of the T66 protein was investigated on resting CD4+ T-cells from HIV-infected individuals under cART in three independent laboratories applying various methods to measure HIV latency reversal. Different positive controls were used within the various latency reversal assays conducted by the three independent laboratories. In Fig. S2, it is demonstrated that the employed T-cell activation positive controls PMA, phytohemagglutinin (PHA), and anti-CD3/CD28 resulted in equipotent HIV reactivation in CD4+ T-cells from three HIV-seronegative donors co-infected with an HIV dual-reporter virus and a lentivirus expressing pro-survival proteins.

**Fig 3:**
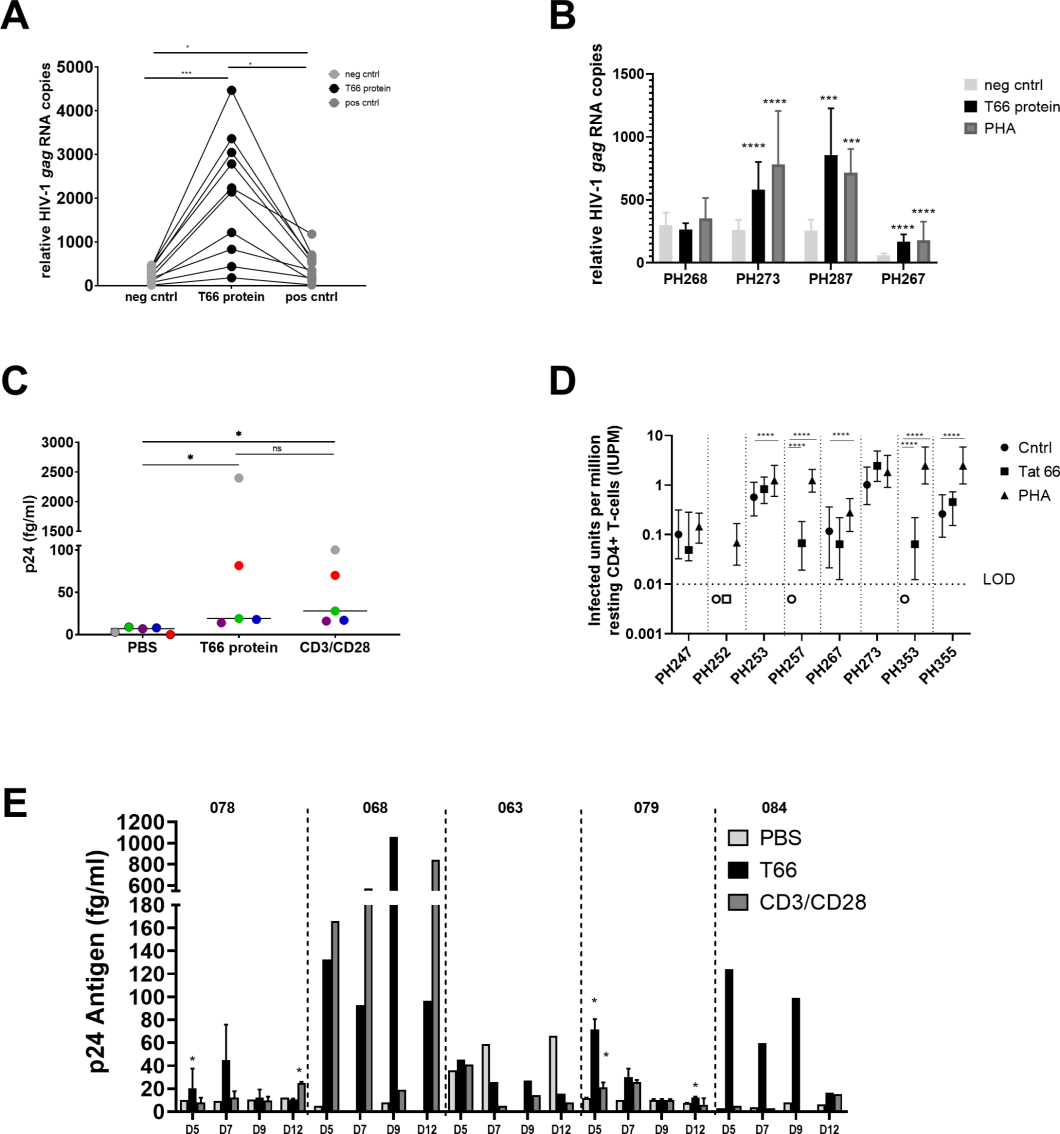
T66 protein displays latency reversal activity in primary cells for combined antiretroviral therapy-treated (cART) donors. (**A**) Production of HIV RNA from CD4+ T-cells: 10 cART-treated donors from cohort A (donor 5 until donor 14) were incubated for 24 hours with T66 protein (black circles), PBS (light gray circles), or PMA (gray). HIV-1 reactivation following latency reversal was assessed by RT-qPCR. Mean of relative HIV-1 gag RNA copies for each donor is plotted on the y-axis (15–40 replicates). For statistical analyses, non-parametric Wilcoxon tests were used. Statistically significant *P* values are represented on the graphs: *P < 0.05; **P < 0.005. (**B**) Production of HIV RNA from resting CD4+ T-cells: four HIV-1 cART-suppressed donors from cohort C (PH268, PH273, PH287, and PH267) were incubated for 24 hours with PBS (light gray bars), T66 protein (4 µM, black bars), or PHA (10 µg/mL, gray bars). HIV-1 RNA production following latency reversal was assessed by RT-qPCR. Relative HIV-1 gag RNA copies are plotted on the y-axis (18 replicates mean ± SD). For statistical analysis, non-parametric Kruskal-Wallis test was used to evaluate statistical significance compared to negative control: ***P = 0.0002, ****P < 0.0001, otherwise not significant. (**C**) CD4+ T-cells from five cART-treated individuals from cohort B (001, 002, 012, 022, and 024 where each donor has his own color) were incubated with T66 protein, anti-CD3/CD28, or PBS. Supernatant was collected on day 10, and p24 production was measured by SIMOA technology. For statistical analysis, non-parametric Kruskal-Wallis test was used to evaluate statistical significance compared to negative control: *P < 0.05, not significant (ns). (**D**) The qVOA assay measures the release of replication-competent HIV. Resting CD4+ T-cells from seven cART-treated suppressed donors from cohort C [PH247, PH252, PH253, PH267, PH353, PH355, one person donated cells on two separate occasions (PH257 and 273) for a total of eight samples] were plated in replicate dilutions of 2.5 (6–24 replicates), 0.5 (6 replicates), 0.1 (6 replicates), and 0.025 (6 replicates) million cells per well, maximally stimulated with a cocktail of PHA (10 µg/mL), 60 U/mL IL-2 and irradiated PBMC (positive control), or activated with T66 protein or 5 U/mL IL-2 (negative control). Cultures were washed and co-cultured with CD8-depleted PBMCs collected from selected HIV-seronegative donors screened for adequate CCR5 expression to expand virus. Culture supernatants were harvested on days 15 and 19 and assayed for virus production by p24 antigen capture enzyme-linked immunoassay (ELISA). Cultures were scored as positive if p24 was detected at day 15 and confirmed at day 19. Each symbol represents the frequency of infected cells after applying maximum likelihood estimation statistics. Error bars represent the 95% confidence interval. For statistical analysis, non-parametric Kruskal-Wallis test was used to evaluate statistical significance compared to negative control: ***P = 0.0002, ****P < 0.0001, otherwise not significant. (**E**) Recovery of replication competent HIV from CD8-depleted total PBMC from five cART-treated individuals from cohort B (078, 068, 063, 079, and 084) were stimulated with PBS (light gray bars), T66 protein (4 µM, black bars), or anti-CD3/CD28 (one bead/cell, dark gray bars). Supernatant was collected on days 5, 7, 9, and 12. p24 production was measured by SIMOA technology. Donors 078 and 079 mean ± SD is plotted. For statistical analysis, non-parametric Wilcoxon test was used to calculate statistical significance compared to negative control: *P < 0.05. The other donors were tested over time as singleton.

The first two studies (Fig. 3A and B) measured cell-associated HIV RNA by reverse transcription-quantitative real-time PCR (RT-qPCR). HIV intracellular RNA was evaluated 24 hours after the addition of the compound. T66 treatment resulted in a significant increase of cell-associated RNA compared to non-stimulated controls (*n* = 10; *P* = 0.0011 negative control versus T66) surpassing HIV RNA levels achieved by PMA stimulation in all 10 participants (Fig. 3A) (*n* = 10; *P < 0.05 medians: 242, 2180, 422 gag RNA copies in the non-stimulated, T66-, and PMA-treated cells, respectively). The concentration of PMA used in this assay was 2.5 times higher than the one reported by Spina et al. (29) and 10 times lower than concentrations stated in other publications (20 – 28). In the second study, T66 treatment increased the levels of cell-associated RNA compared to the non-stimulated condition in CD4 T-cells from three out of four cART-treated individuals. The cell-associated RNA levels were comparable to the levels observed following PHA stimulation (Fig. 3B). To evaluate virion release due to HIV-1 reactivation, p24 secretion in the supernatant was assessed by the ultrasensitive SIMOA (Fig. 3C and E). CD4+ T-cells were isolated from five cART-treated individuals and were incubated for 10 days with either T66 protein or anti-CD3/anti-CD28 antibodies. Both treatments led to similar p24 induction in all five donors. An additional study using a similar methodology confirmed T66-mediated p24 induction in CD8-depleted peripheral blood mononuclear cell (PBMC) collected from five HIV-infected individuals following a 12-day stimulation (Fig. 3E). Extracellular p24 was induced in all samples after the addition of T66 protein resulting in similar or increased p24 levels with respect to anti-CD3/CD28-treated samples. Due to donor variability, peak p24 production as well as time to reach maximum virus release differed across donors. Finally, the quantitative viral outgrowth assay (qVOA) was applied to investigate the capacity of the T66 protein at inducing the expression of replication competent proviruses (*n* = 6 cART-treated individuals, Fig. 3D). In this assay, T66 treatment showed significant donor-to-donor variability in its ability to induce outgrowth of replication-competent virus. Three donors (PH247, 252, and 273) were not assessable in qVOA due to low infectious units per million and/or minimal response to PHA activation. Cells from three donors (PH253, 267, and 355) did not release a significantly more replication-competent HIV following T66 treatment compared to control, and two donors released a significantly more replication-competent HIV following T66 treatment compared to control (PH257 and PH353). To conclude, T66 protein in many instances worked as well or better than field-established positive control stimulators of HIV-1 gene expression and, therefore, is a promising candidate for the reversal of latency in CD4+ T-cells of cART-treated individuals.

### T66 minimally perturbs the transcriptome and does not induce global T-cell activation

**Fig 4:**
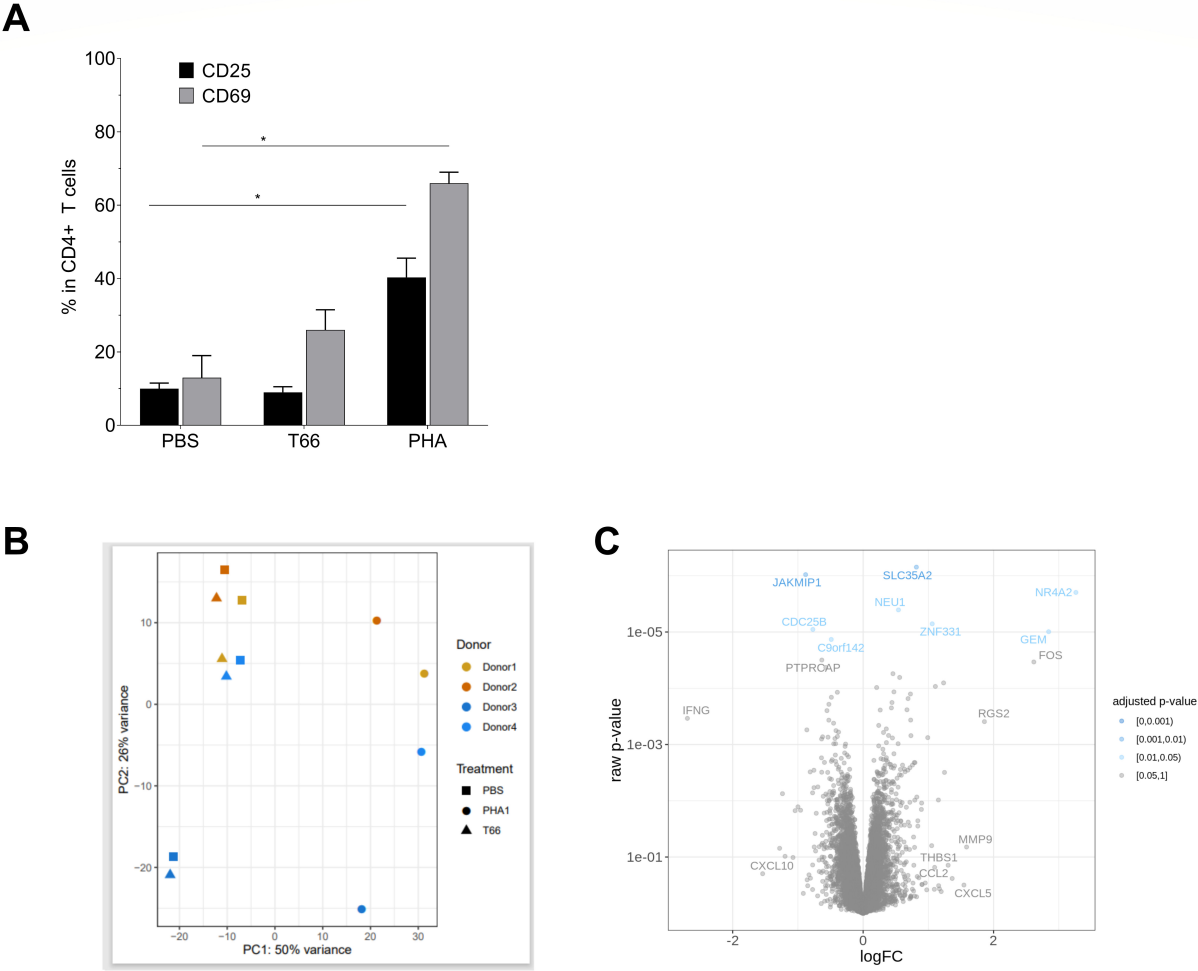
T66 protein does not induce activation of T-cells and has minimal effect on transcriptome. (**A**) CD4+ T-cells from four cART-treated individuals from cohort A (donor 1 until donor 4) were exposed for 24 hours to PBS, T66 protein (4 µM), or PHA (10 µg/mL). Expression of CD25 and CD69 was assessed by flow cytometry. Percentage of positive cells (gray bars) and CD25-positive cells (black bars) are plotted. For statistical analyses, non-parametric Kruskal-Wallis test was used to evaluate statistical significance: *P < 0.05 (**B**) 24 hours after stimulation cells were lysed to isolate RNA and to evaluate effects on transcriptome by microarray. Principal component analysis (PCA) plot is shown for four donors and three different treatments [PBS, T66 (4 µM), PHA (10 µg/mL)]. (**C**) Volcano plot comparing differentially expressed genes between PBS-treated and T66-treated CD4+ T-cells. Statistically significance is based on Benjamini-Hochberg adjusted *P*-value <0.05.

Clinically acceptable LRAs should not induce global T-cell activation (75). In contrast to the well-known mitogen PHA, T66 protein did not induce global T-cell activation, as shown by the absence of CD69 (early activation marker) and CD25 (late activation marker) up-regulation (Fig. 4A). Moreover, micro-array experiments were conducted on total RNA extracted from CD4+ T-cells from four cART-treated individuals to study the impact of the T66 protein on the transcriptome of the cells. PBS- and PHA-treated samples were included as negative and positive controls, respectively. To ensure that T66 was present and active in the HIV-infected CD4+ T-cells, latency reversal was confirmed in those samples by HIV RNA quantitative real-time PCR (qPCR) (Fig. S3). The transcriptome profile from microarray experiments revealed that T66-treated cells cluster with PBS-treated cells for all participants, suggesting no significant impact of T66 treatment on the host transcriptome (Fig. 4B). This is in sharp contrast with PHA-stimulated cells which formed a second cluster, confirming the profound impact of the mitogen PHA on the human transcriptome. Based on volcano plots, we identified eight genes as differentially expressed between PBS-treated samples and T66-treated samples (adjusted *P* < 0.05, Fig. 4C). However, for these eight differentially expressed genes, the log2 fold change was lower than 2, indicating that treatment with the T66 protein minimally perturbs the transcriptome of CD4+ T-cells.

### T66 mRNA exceeds the latency reversal levels achieved with T66 protein in T-cell lines

**Fig 5:**
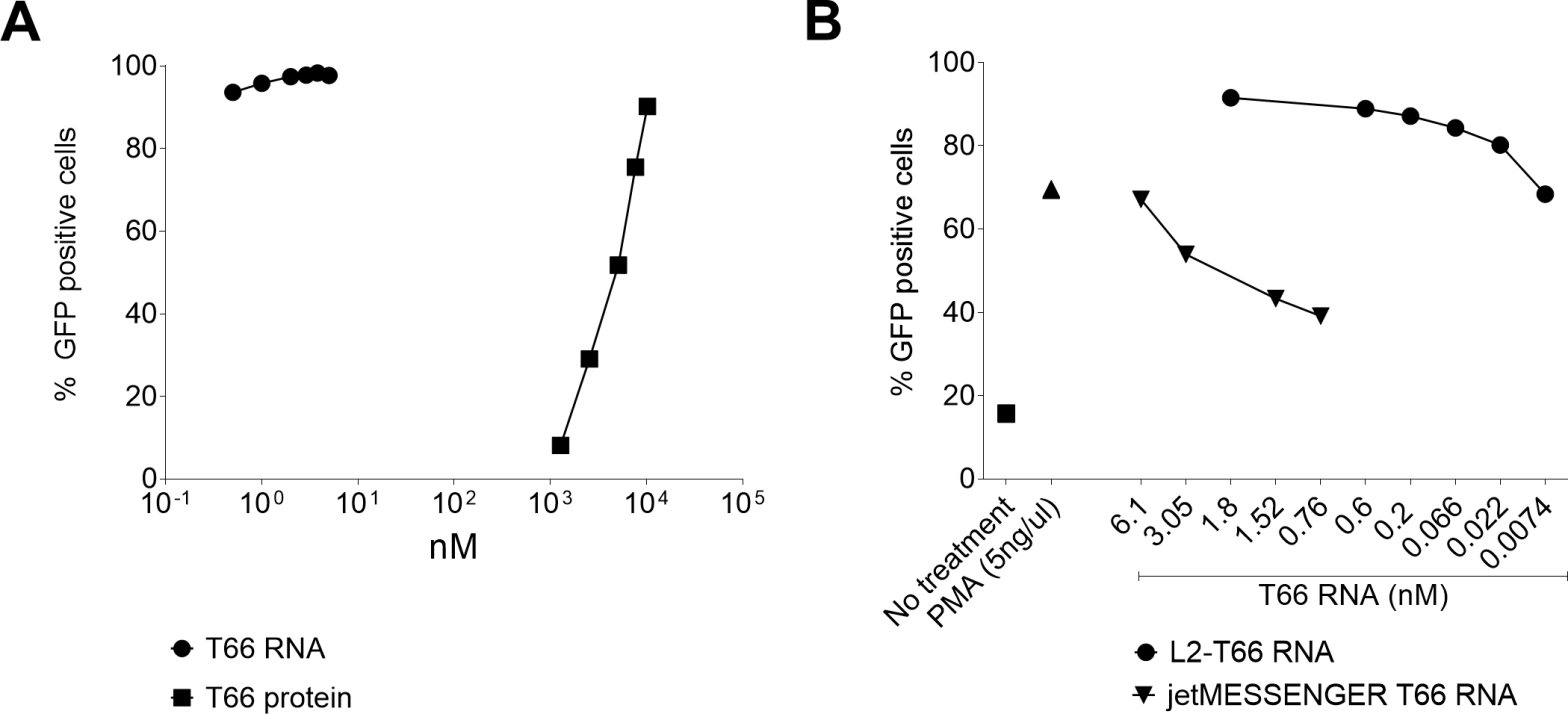
Reactivation of latently infected cell lines by T66 RNA. (**A**) MT-4LTR-GFP cell lines were treated with a dose response of T66 mRNA (circles) delivered by jetMESSENGER or with a dose response of T66 protein (squares). Twenty-four hours post stimulation LTR activation was assessed by measuring the percentage of GFP-positive cells. Fold change compared to untreated MT-4 LTR-GFP cells is plotted. (**B**) In Jurkat cl50 cells, two delivery methods of T66 RNA were evaluated: jetMESSENGER (triangle) or LNP-2 (circles) to reactivate. LTR activation is expressed as percentage of GFP-positive cells.

T66 protein showed robust latency reversal in multiple assays performed with different methodologies and in different laboratories, while neither global T-cell activation nor any significant transcriptome perturbation was observed. However, significant amounts of protein will be required for *in vivo* administration of T66 protein to achieve maximum HIV activation, due to limitations in cellular protein transduction and substantial endosomal trapping of the T66 protein. One strategy to address this potential liability is to use T66 mRNA delivered by lipid nanoparticles which would result in enhanced cytosolic protein exposure compared to externally applied T66 protein. Initially, a variety of commercially available reagents were evaluated for the transfection of T66 mRNA into the T-cell lymphoma MT-4-LTR-GFP cell line. Among all the different methods tested, jetMESSENGER was the most efficient transfection reagent (data not shown). Next, the latency reversal capacity of jetMESSENGER-delivered T66 mRNA was compared with the administration of the T66 protein (Fig. 5A). The performed dose-response experiment showed that T66 mRNA-induced LTR activation as measured by GFP expression reached an upper plateau at the lowest concentration tested (0.5 nM), whereas T66 protein treatment required 4 µM to achieve maximum activation. We also evaluated various lipid nanoparticle (LNP) formulations for efficient delivery of the T66 mRNA and identified LNP-2 as a promising LNP formulation (data not shown). The LNP-2 formulation was compared to jetMESSENGER formulation in the Jurkat clone 50 cell line (Fig. 5B). While 6.1 nM jetMESSENGER/T66 mRNA led to a similar GFP induction as PMA [67% vs 69% GFP (+)], 1.8 nM LNP2-T66 mRNA enhanced the population of GFP (+) cells to 91.5%. Given that the clone 50 cell line was generated via multiple rounds of PMA-stimulated GFP (+) cell sorting, it was striking to see that the LNP-T66 mRNA delivery exceeded PMA-induced GFP activation. It is equally remarkable that the lowest tested LNP-2-T66 mRNA concentration (7 pM) resulted in similar activation (68.4%) as the jetMESSENGER-T66 mRNA formulation (67.1%) at its highest concentration (6.1 nM) (Fig. 5B). This translates to a potency increase of the LNP-2 formulation over the jetMESSENGER reagent by more than 800-fold.

### LNP delivery of the T66 mRNA induces potent HIV reactivation in CD4+ T-cells from cART-treated individuals

**Fig 6:**
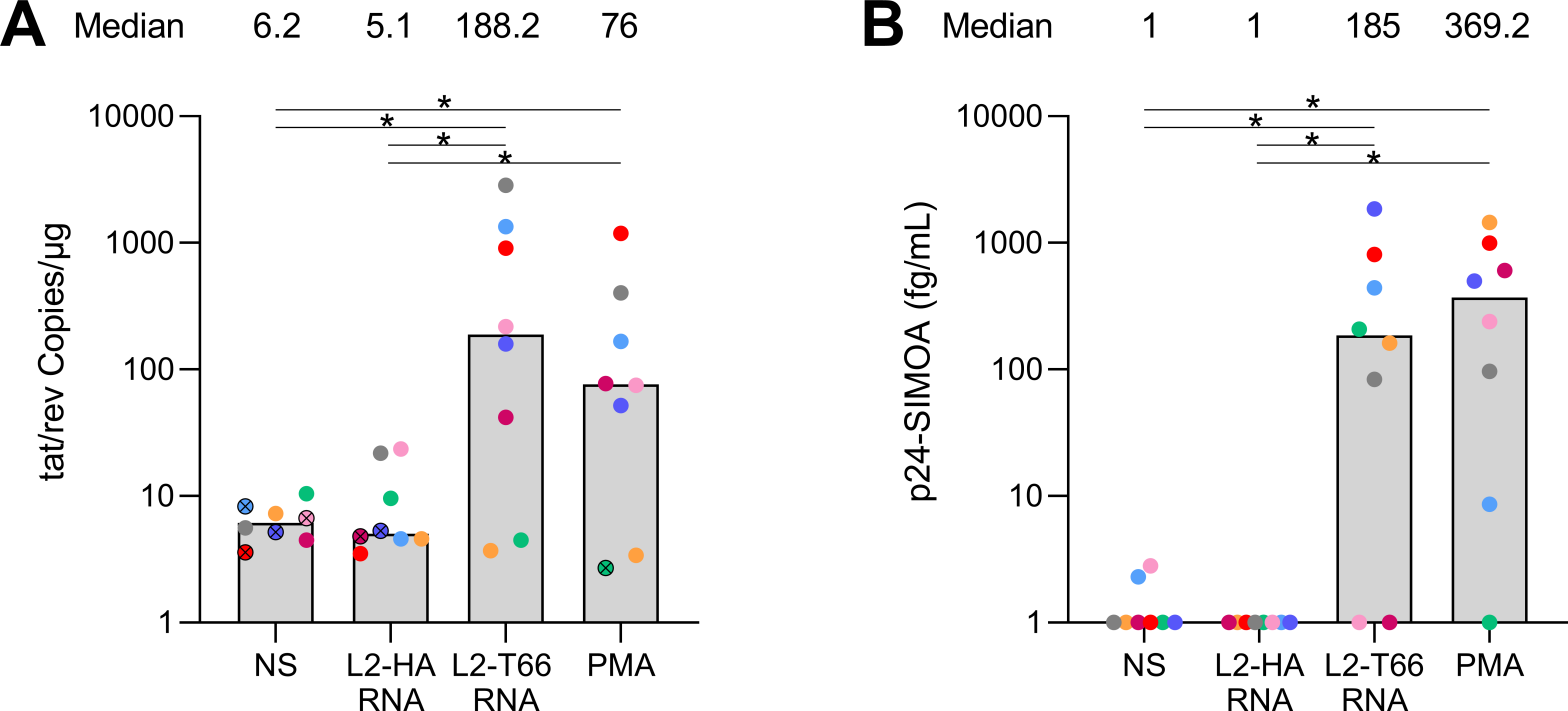
Reactivation of latent-infected CD4+ T-cells from HIV-infected patients. (**A**) CD4+ T-cells from eight different cART-treated individuals from cohort D were treated for 48 hours with PBS (NS), HA-LNP2, T66 mRNA-LNP2 (Tat LNP2), or PMA as positive control. RNA was extracted from the cells, and RT-ddPCR was performed to quantify tat/rev transcripts. Results are expressed as number of *tat/rev* copies per microgram of RNA. Undetectable measures are represented by the following symbol: ⨂. The limit of detection (LOD) assumes that only one positive droplet was detected over the three replicates and is calculated using Poisson modeling: LOD = −LN(1 − (*P*/t)) * (1000/0.91), where *P* = 1 for the number of positive droplets, t = total number of droplets across the three replicates, and 0.91 corresponds to the partition volume. (**B**) Viral release was evaluated by measuring p24 secretion in supernatant by SIMOA technology. For statistical analyses, non-parametric Wilcoxon tests were used. Statistically significant *P* values are represented on the graphs. *P < 0.05.

To confirm our findings obtained in cell lines in a more physiologically relevant model, we tested the latency reversal capacity of the T66 mRNA when delivered through the LNP-2 formulation (L-2-T66 RNA) in CD4+ T-cells from eight cART-treated individuals. A 48-hour stimulation with PMA (10 nM) was used as positive control, while two negative controls were used: (i) non-stimulated cells (NS) and (ii) a lipid nanoparticle containing the hemagglutinin A peptide from influenza (L2-HA RNA). Reactivation capacity was assessed by (i) droplet digital PCR to quantify the levels of multiply spliced tat/rev mRNAs (Fig. 6A) and (ii) p24-ultrasensitive SIMOA in the supernatant as a proxy for viral particle release (Fig. 6B). While low levels of tat/rev RNA copies were observed in the negative control conditions (medians, 6.2 and 5.1 copies/µg in NS and L2-HA RNA conditions, respectively), the number of tat/rev RNA copies was significantly increased following L2-T66 RNA and PMA stimulation compared to the negative control conditions (*P* = 0.04) (Fig. 6A). Although not significant, L2-T66 RNA tended to induce higher levels of latency reversal when compared to PMA [medians, 188 and 76 copies/µg in T66- and PMA-treated cells, respectively (*P* = 0.25)]. In negative control samples with low or undetectable p24 levels, L2 T66 RNA and PMA induced detectable concentrations of p24 in 6/8 and 7/8 participants, respectively (Fig. 6B). While levels of induced p24 in the supernatant were lower in the L2 T66 mRNA condition compared to the PMA-treated cells (medians, 185 and 369 fg/mL in the L2 T66 RNA- vs PMA-treated cells, respectively), those differences were not statistically significant (*P* = 0.84). In conclusion, the T66 mRNA delivery by the LNP-2 formulation is highly efficient at inducing latency reversal in CD4+ T-cells from cART-treated individuals.

## DISCUSSION

While cART has greatly improved the quality of life of people living with HIV (76), it does not lead to cure (elimination of the virus from its host). The virus persists in a latent and largely transcriptionally silent state in a small pool of memory CD4+ T-cells (3, 9) invisible to the immune system. cART treatment interruption studies result in rapid viral rebound in the vast majority of long-term-treated HIV-infected individuals (77). The latently infected cellular reservoir represents the major roadblock to viral cure. In this paper, we identified a novel agent T66, which is highly efficient at inducing latency reversal, both in cell lines and in primary CD4+ T-cells isolated from cART-treated individuals.

HIV-1 Tat protein is probably the strongest transcriptional transactivator known to date (78, 79). Tat is encoded by two exons: the first exon (72 aa) comprises all essential functional regions and is highly conserved, while the second exon (86–101 aa) codes for a structurally non-defined variable C-terminal region with unknown functionality. The transcriptional effect of Tat is initiated by the binding to TAR, a stem-loop structure in the nascent transcript resulting in the recruitment of the p-TEFb complex to the HIV-1 LTR promoter. This interaction leads to the phosphorylation of RNA polymerase II (RNAPII) and other associated factors to engage in the production of full-length HIV RNA transcripts from a paused transcription complex (80). More recently, it was discovered that p-TEFb also promotes binding of additional elongation factors such as AFF4, ENL, and AF9 to form an assembly called “super elongation complex” explaining the incredible transactivation power of Tat (81 – 83). In the absence of Tat, RNA polymerase II initiates transcription from the LTR promoter by typically stalling downstream of the TAR, producing short, abolished transcripts (84, 85). The ability of Tat to strongly activate viral transcription has long been known, but few attempts have been made to harness the protein as an LRA. The general concern to explore Tat as LRA likely stems from the reported off-target activity and cellular toxicity exerted by full-length Tat (19, 86 – 88). Despite these reports, TAR-independent effects remain controversial. Transgenic mice expressing full-length Tat did not develop any phenotype other than reduced glutathione levels (89) or atypical skin lesions after 2 years of age (90). A more recent study confirmed a total lack of side effects in transgenic mice secreting Tat into the serum (89). However, to reduce the risk of potential non-specific receptor engagement by extracellular Tat, we focused on the identification of the smallest variant retaining potent Tat-72 transactivation activity by deletion of the Tat C-terminus which contains the non-structured region of the protein. We have shown that Tat-66 is the smallest Tat variant that maintained equipotent LTR activation when compared to full-length Tat.

The reactivation capacity of the T66 protein was assessed in different cell lines as well as in primary CD4+ T-cells from HIV-infected individuals. The capacity of T66 to reverse HIV latency was shown by three independent laboratories (J&J in collaboration with ITM, Hospital Pitié Salpetière, and UNC HIV Cure Center) with three different read-outs (cell-associated RNA, p24-SIMOA, and qVOA) (Fig. 3). The cell-associated RNA assay probes for proviral transcriptional activation, the ultrasensitive SIMOA assay confirms that a structural virion protein has been translated and released, and the qVOA assay of resting CD4 T-cells from individuals living with HIV on cART treatment rigorously explores whether replication competent HIV has been produced *ex vivo* by a persistently infected cell.

The T66 protein induced consistently higher levels of cell-associated HIV RNA than the positive control PMA in all tested donors, while it resulted in similar p24 supernatant levels when compared to CD3/CD28 T-cell co-stimulation. However, in the qVOA assay, virus was recovered with somewhat greater frequency following mitogen stimulation than T66 treatment. Given the significant heterogeneity in HIV inducibility across different donors generally observed in viral outgrowth assays after LRA exposure, further studies with more donors will be needed to fully assess the *in vitro* latency reversal activity of T66. The strength of this study is that HIV latency reactivation potential of T66 is shown by three independent laboratories. However, a caveat of the study is that in each laboratory, different positive controls were used, which make head-to-head comparisons more difficult. Of note, the three positive controls showed equipotent HIV reactivation in primary CD4 T-cells from three different donors that were co-infected with dual-reporter virus (Fig. 2). This is the first time to identify a molecule tested in different HIV latency assays that demonstrates robust induction of HIV expression and latency reversal similar to PKC modulator PMA and importantly in the absence of any CD4 T-cell activation (29). The promising activity of T66 merits further testing in combination with other LRAs or immunotherapies *ex vivo* as well as *in vivo* models.

A major barrier to considering Tat as LRA is based on the reported off-target activities and toxicities using recombinant full-length Tat86 protein produced in bacteria. Two strategies were applied to address this liability: chemical protein synthesis was chosen over recombinant protein production to avoid any biological contamination with TLR ligands that could lead to pattern recognition receptor activation of signaling pathways unrelated to Tat-TAR-mediated transactivation. Secondly, a large part of the C-terminus was deleted to remove any potential binding sites of Tat to cellular receptors which may be present in this part of the protein such as the RGD motif (residues 78–80) that interferes with some integrin receptors (91, 92).

A major clinical challenge will be to deliver sufficient T66 protein to HIV-infected CD4+ T-cells *in vivo*. Administration of a substantial amount of protein may be required due to inefficient cellular protein transduction, significant endosomal trapping, high plasma protein binding, and potential neutralization by preexisting anti-Tat antibodies. One strategy to address these possible protein-associated limitations would be to deliver T66 mRNA packaged into nanoparticles to CD4+ T-cells. A proof-of-concept study *in vitro* was initiated to evaluate whether T66 mRNA would induce similar HIV reactivation as observed with the T66 protein. Most notably, T66 mRNA transfected with jetMESSENGER into an MT4 HIV-GFP reporter cell line exceeded the activation of T66 protein by more than 4 log_10_. Maximal reporter expression was achieved with 0.5 nM T66 mRNA, while it required 5 µM of protein to reach similar activation. Finally, the ability of T66 mRNA to induce latency reversal in a difficult to reactivate Jurkat clone 50 cell line was assessed: 6 nM of T66 mRNA transfected with jetMESSENGER showed equipotency with PMA. The recognition of superior T66 mRNA activity led to test additional engineered LNPs for efficient mRNA delivery into primary lymphocytes. LNP-2 was selected as the lead LNP that significantly exceeded the level of reactivation observed with the jetMESSENGER formulation. The LNP-2 formulation was, therefore, further explored in CD4+ T-cells from HIV-infected individuals, where PMA and L2 T66 mRNA induced similar levels of tat/rev mRNA and released similar levels of p24 in the supernatant.

In conclusion, our work suggests that T66 mRNA shows similar potency to the gold standard activation compound PMA to induce latency reversal and viral particle release. In contrast to PMA, T66 exposure to primary CD4+ T-cells led to neither T-cell activation nor showed any significant perturbation of the human transcriptome. Therefore, T66 protein or mRNA could be an important LRA to be considered for HIV cure combination regimens. Clinical translation of our approach will require validation of the L2-T66 mRNA latency reversal capacity and an assessment of pharmacokinetic characteristics and compound-induced toxicity of Tat in a relevant preclinical model.

## MATERIALS AND METHODS

### Participants and blood collection

In this study, patients living with HIV on stable suppressive cART were recruited from four different institutes: Institute of Tropical Medicine (Antwerp, Belgium; *n* = 14; cohort A), Hospital Pitié Salpetière (Paris, France; *n* = 10; cohort B), University of North Carolina HIV Cure Center (United States; *n* = 9, cohort C), and HIV Cure Research Center (Ghent, Belgium; *n* = 8, cohort D). HIV-infected individuals included in this study were all on cART for at least 1.5 years and had a viral load <20 copies/mL. Patient characteristics are summarized in Table 1. PBMCs from healthy donors were obtained from the Red Cross (Mechelen, Belgium).

**TABLE 1:** Patient characteristics

Laboratory	Cohort A (*n* = 14)^*a*^	Cohort B (*n* = 10)^*b*^	Cohort C (*n* = 9)^*c*^	Cohort D (*n* = 8)^*d*^
Age in years, median, (IQR^*e*^)	52 (34–69)	50 (34–58)	56 (33–59.8)	52 (48–62)
CD4 in cells/mm^3^, median (IQR)	821 (361–1113)	628 (462-689)	664 (473–789)	823 (335–126)
CD8 in cells/mm^3^, median (IQR)	1113 (369–1689)	876 (643–10007)	604 (426–812)	963 (567–1800)
CD4/CD8 ratio, median (IQR)	0.67 (0.31–2)	0.7 (0.6–1)	0.98 (0.84–1.65)	1 (0.5–1.3)
CD4 nadir in cells/mm^3^, median (IQR)	197 (83–285)	241 (209–300)	419 (157–551)	291 (98–488)
Years since first diagnosis, median (IQR)	17.3 (3.6–24)	19 (25–10)	5 (4–7)	16 (8.4–30.1)
Years on cART, median (IQR)	15.7 (3.4–17)	7.2 (5.1–11.0)	4.92 (3.43–6.42)	12 (1.2–24.3)
Viral load log10 (copies/mL)	<20	<20	<20	<20
Years suppressed, median (IQR)	12 (1.2–24.3)	7.2 (5.1–11)	3.74 (2.288–6.18)	15.7 (3.4–17)

^*a*^ Labeled as donor 1 to donor 14.

^*b*^ Labeled as 001, 002, 012, 022, 024, 078, 068, 063, 079, and 084.

^*c*^ Labeled as PH268, PH273, PH287, PH267, PH247, PH252, PH253, PH257, PH353, PH355. PH257, and PH273 are the same participant, but two different donations.

^*d*^ Labelled as MRC02, MRC04, MRC08, MRC11, MRC15, MRC21, MRC24, MRC25.

^*e*^ Interquartile range.

### Generation of reporter cell lines to measure latency reversal activity

HEK293T cells (ATCC, Manassas, USA) were transfected with pNL-EGFP/pVSV using polyethylenimine (PEI; Polysciences, Germany) (PEI/DNA 6/1 ratio; 500 µL/well). Forty-eight hours post transfection virus was collected and concentrated with PEG Virus Precipitation Kit (BioVision, Inc., USA) according to the manufacturer’s instructions. The Jurkat Clone E6.1 (ATCC, USA) cell line was then infected with PEG concentrated virus (1/10 ratio) for 24 hours. After removing the virus, the cells were cultured for 1 week. The GFP (−) cell population potentially harboring latent virus was sorted and stimulated with 10 ng/mL PMA (Sigma Aldrich) followed by sorting the GFP (+) population. After 1 week, the GFP-negative cells were sorted again and restimulated with 10 ng/mL PMA. This process was repeated four times. Finally, GFP (+) were single cell sorted and cultured for 3 weeks. After this process, two clones were selected with low GFP background, Jurkat E6.1 clone 32, and Jurkat E6.1 clone 50), which contain latent HIV with a GFP reporter.

To generate MT-4-LTR-EGFP cells, MT-4 cells [gift from Nakashima et al. (93)] were transfected with a plasmid containing LTR-driven EGFP expression plasmid (Clontech Laboratories, Inc., USA) and pFX2 (Invitrogen Corporation, USA) in Opti-Mem Reduced Serum Media (Thermo Fisher Scientific Europe) according to the manufacturer’s instructions. Cells were selected based on geneticin (Thermo Fisher Scientific, Europe) resistance. To obtain a stable cell line, cells were sorted based on high viability and low GFP background.

### Generation of plasmids

The full-length Tat protein (86–101 aa) is translated from two different exons where exon-1 (1–72 aa) contains all domains essential for transactivation. The following Tat coding sequences (based on the molecular HIV clone NL4.3) were introduced into the DNA expression vector pVax-1 (Life Technologies): tat86 (86 referring to length in amino acids) *tat*72, *tat*70, *tat*68, *tat*66, *tat*65, *tat*64, *tat*60, *tat*57, and *tat*2-72 (which lacks the N-terminal second amino acid after methionine). The different Tat mutants were generated by C-terminal deletions. The HIV-1NL4.3 LTR was cloned into pG5luc (Promega) upstream of the firefly luciferase gene, yielding the plasmid pLTR-FLuc. The transfection normalization plasmid pEF-RLuc was generated by swapping the CMV promoter with the elongation factor 1α promoter in pcDNA3.1 followed by the downstream introduction of the Renilla luciferase coding sequence in the multiple cloning site.

### Dual luciferase assay for Tat activation assessment

HEK293T-cells were co-transfected with the same amount (100 ng per transfection) of the different *tat* expression plasmids, the pLTR-FLuc (50 ng/transfection) and the pEF-RLuc (50 ng/transfection) plasmids. The Dual Glo-reporter assay (Promega) was used to measure LTR activation via the Firefly luciferase read-out along with signal normalization by Renilla luciferase. LTR activity was expressed as percentage of wild-type exon-1 *tat*72 activity.

### Production of the T66 protein

The Tat66 (T66) protein was synthesized as trifluoroacetate salt by Bachem. The provided lyophilized powder was resuspended in sterile water containing 1 mM DTT to a final concentration of 1 mg protein/mL. The pH was adjusted with NaOH to pH 7.4.

### HIV reactivation in cell lines

MT-4-LTR-GFP, Jurkat E6.1 clone 32, and clone 50 were incubated overnight with a dose range of the T66 protein or T66 mRNA. Medium was used as a negative control, while PMA (10 ng/mL), CD3/CD28 dynabeads (1 bead per cell, Thermo Fisher, Europe), or PHA (10 µg/mL; Sigma Aldrich) were used as positive controls. LTR activation was measured by flow cytometry (BD Fortessa, BD Biosciences, Erembodegem, Belgium) based on GFP expression.

### HIV reporter assay on primary CD4+ T-cells

PBMCs were isolated from HIV-negative blood donors by Ficoll gradient centrifugation. CD4+ T-cells were purified by positive selection using Miltenyi magnetic beads (Miltenyi Biotec, Gladbach, Germany). These cells were stimulated with anti-CD3/CD28 magnetic dynabeads (1 bead per cell, Thermo Fisher, Europe) for 2 days. Following the removal of the beads, cells were co-infected with a single cycle HIV dual reporter virus expressing GFP and mouse CD48 and a lentiviral vector coding for pro-survival proteins MCL1 and Bcl2 co-expressing cell surface mouse CD24. Infected cells were cultivated in the presence of IL-2 (10 IU/mL, Roche, Basel, Switzerland). Three days after coinfection, cells were sorted based on expression of mouse CD48 and mCD24 (BD Biosciences, Belgium). These cells were further cultivated for another 7 days on IL-2 containing medium and rested for 3 extra days in assay medium (RPMI, 10% fetal calf serum [FCS]). To evaluate HIV reactivation, cells were plated at a concentration of 2 million per milliliter and incubated in the presence of T66 protein (4 µM), negative control (PBS), or positive control PMA (5 ng/mL) for 24 hours. Reactivation capacity was evaluated by measuring the percentage of GFP+ cells using flow cytometry (Fortessa, BD Biosciences, Belgium).

### *Ex vivo* reactivation of HIV measured by RT-qPCR

CD4+ T-cells were isolated from 200-mL whole blood donated by cART-treated individuals, using CD4 microbeads (Miltenyi Biotec, Germany) according to manufacturer’s protocol. Resting CD4+ T-cells were isolated from leukapheresis products as previously described (94). Ten to twenty replicates of cell pools plated at 1 million total or resting CD4+ T-cells per well were incubated overnight in media (RPMI, 10% FCS) with the T66 protein (4 µM), negative control (PBS), or positive control (PMA, 5 ng/mL or highly purified PHA 10 µg/mL Thermo Fisher, Europe). Total RNA was isolated using the MagMax 96 total RNA isolation kit (Ambion, Europe) following the manufacturer’s protocol. Reverse transcription was performed using the Superscript III First Strand synthesis kit (Invitrogen, Europe) according to the manufacturer’s protocol. qPCR was conducted on the cDNA using gag-specific primers (GGACCAAAGGAACCCTTTAGAGA; GGACCAACAAGGTTTCTGTCATC) in the presence of nucleic acid dye SYBR Green (Invitrogen, Europe). Standard curves were generated using cDNA synthesized from *in vitro* transcribed RNA. The detection limit of the qPCR was determined to be at 10 copies per reaction. Relative HIV-1 gag RNA copies were calculated related to the standard curve.

### *Ex vivo* reactivation of HIV measured by p24 detection

PBMCs were isolated from whole blood of cART-suppressed individuals by Ficoll gradient centrifugation. CD4+ T-cells were purified from PBMCs by negative selection (EasySep Human CD4+ T-cell Isolation Kit, Stem Cell, Cambridge, UK). CD4+ T-cells were plated in 200 µL of medium (RPMI, 10% human serum) containing IL-2 (Roche, 10 IU/mL) and IL-7 (Peprotech, 1 µg/mL) in triplicate (200,000 cells/well). The T66 protein was added to a final concentration of 4 µM. Anti-CD3/CD28 antibodies were used as a positive control, while PBS was used as a negative control. Cells were incubated at 37°C for 10 days, and medium was changed every 3–4 days (180 µL of supernatant removed and refreshed with new medium supplemented with IL-2 and IL-7). Supernatant was frozen at −80°C for subsequent detection of p24 by SIMOA (Quanterix, USA). In another set of experiments, CD8 T-cells were depleted from the PBMC by positive selection (EasySep CD8 T cell isolation kit, Stem Cell, Cambridge, UK). CD8-depleted PBMCs were cultured in medium (RPMI, 10% human serum) and treated as described above for the CD4+ T-cells. Frozen supernatant from above cultures was thawed, and p24 production was evaluated by SIMOA following the manufacturer’s instructions.

### *Ex vivo* reactivation of HIV measured by qVOA

The qVOA assay was performed as previously described (11, 94). Briefly, for each donor and for each treatment condition, 20–60 million resting CD4+ T-cells were used to set up the qVOA assay. The qVOA assay was set up in replicate wells in limiting dilutions (2.5, 0.5, 0.1, and 0.025 million cells per well). The range in the number of input cells is selected (based on prior studies of each donor) so that some cultures are positive at the highest number of input cells and that cultures are rarely positive at the lowest number of input cells. The cells are then activated with PHA (Remel, Thermo Fisher, USA) in the presence of a fivefold excess of allogeneic irradiated PBMCs from an HIV-seronegative donor and 60 IU/mL IL-2 or T66 protein (4 µM) or 5 U/mL IL-2 (controls) for 24 hours. Afterwards, cells were co-cultivated with CD8-depleted PHA blasts. Culture supernatants were harvested on day 15 and assayed for virus production by p24 antigen capture ELISA (ABL, Rockville, MD, USA). Limiting dilution statistics were used to calculate the point estimate of infected cell frequency, reported as infectious units per million of CD4+ T-cells (95, 96).

### Transcriptome analysis by micro-array

Bulk CD4+ T-cells isolated from cART treated individuals were treated with T66 protein (4 µM), PHA (10 µg/mL), or PBS. Twenty-four hours later, 100,000 cells were transferred to a tube containing 100 µL of RLT buffer (Qiagen, Hilden, Germany). Cells were stored at –80°C until further processing. RNA extraction was prepared with the RNeasy plus mini kit (Qiagen, Germany). Amplification and labeling of total RNA were performed using the GeneChip PICO Reagent Kit following the manufacturer’s protocol (Thermo Fisher, Europe). Biotin-labeled target samples were hybridized to the Clariom GO Screen containing probes for over 20,000 genes. Target hybridization was processed on the GeneTitan Multi-Channel Instrument (Applied Biosystems, Waltham, USA) according to manufacturer’s instructions provided for expression array plates. Images were analyzed using the GeneChip Command Console Software (Thermo Fisher, Europe). The microarray data set was background corrected and normalized with robust multiarray analysis (38) and summarized with the ClariomSHumanHT_Hs_ENTREZG v21.0.0 chip definition files (39). Quality assessment was performed with array QualityMetrics (97). Using the expression values for all 22,593 probe sets, we evaluated the main source of variability contained in the data set using principal component analysis. We performed differential gene expression by comparing different treatments) using linear regression models for microarray data analysis (Limma) (98). The data are available in GEO database (accession number GSE235229).

### Evaluation of global T-cell activation by flow cytometry

Following treatment with the T66 protein (4 µM), PHA (10 µg/mL), or the negative control (PBS) for 24 hours, CD4 T-cells were stained with anti-CD25 PerCp-cy5.5 and anti-CD69 APC (BD Biosciences, Belgium) for 15 min at 4°C. Cells were analyzed on Fortessa (BD Biosciences, Belgium).

### Generation of T66 mRNA

A DNA template for the *in vitro* transcription of mRNA was generated by introducing the following elements in a standard pDNA3.1 vector (Thermo Fisher, Europe): a T7 promoter, followed by 5′- and 3′-UTRs from either the human α-globin or the frog (Xenopus) α-globin, terminated by a 120-nt poly-A tail. Downstream of the Poly-A tail is a unique restriction enzyme site for linearization. In reactions using the capping reagent AG CleanCap (Trilink, San Diego, USA), the native T7 promoter sequence was changed to TAATACGACTCACTATAAG. An HIV-1 T66 codon-optimized gene sequence was introduced via restriction enzyme-based cloning into the DNA plasmid between the 5′-UTR and 3′-UTR. Sequence-verified DNA constructs were scaled up, linearized, and column purified with the PureLink Gel Extraction Kit (Thermo Fisher, Europe). *In vitro* transcription was performed with either HiScribe T7 ARCA mRNA kit or the HiScribe *In Vitro* Transcription Kit (NEB) according to the manufacturer’s recommendation. A 500-ng linearized plasmid DNA (pDNA) was added per 20-µL transcription reaction. The ribonucleotide uridine was, in some cases, replaced with N1-methypseudouridine. The capping reagent AG Cleancap (Trilink, USA) was added at 5 mM concentration to the transcription reaction along with 0.1 U of inorganic pyrophosphatase YIPP (NEB). *In vitro* transcription was typically performed for 3 hours at 37°C. The *in vitro* transcript was purified by column purification using RNA miniprep/maxiprep kits (Qiagen, Germany) according to the provided protocol. The RNA was quantified with the Nanodrop spectrophotometer, and its integrity was verified by agarose gel analysis.

### Identification of nanoparticle formulations for efficient T66 mRNA delivery

A panel of commercial transfection reagents were tested in a cellular HIV-GFP reporter assay to identify the optimal delivery system of T66 mRNA into Jurkat T lymphocyte cells: JetPEI (Polyplus, Illkrich-Grafenstaden, France), jetMESSENGER (Polyplus, France), Viromer (Lipocalyx, Halle, Germany), TransIT mRNA (Mirus, Marietta, USA), RNAiMax (Thermo Fisher, Europe), and Messenger Max (Thermo Fisher, Europe). The transfections were performed with 1 × 10^5^ Jurkat cells plated in 100-µL medium in 96-well plates using 100 ng/mL T66 mRNA formulated with the different reagents according to the supplier’s instructions.

LNPs were prepared by mixing a lipid/ethanol solution with an aqueous RNA solution. Specifically, lipid excipients (ATX—Arcturus Therapeutics proprietary ionizable lipid, phospholipid, cholesterol, and polyethylene glycol derivatized lipid) were dissolved in ethanol. An aqueous solution of the RNA was prepared in citrate buffer (pH 3.5). The lipid mixture was then combined with the RNA solution using a NanoAssemblr microfluidic system (Precision NanoSystems). Nanoparticles thus formed were dialyzed against HEPES buffer (pH 8.0) using dialysis tubing (Repligen, Waltham, USA) at room temperature. The concentration of the formulation was adjusted to the final target RNA concentration using Ultra centrifuge concentrator tubes (Millipore Sigma, Burlington, USA) and was sterile filtered. Post filtration, bulk formulation was aseptically transferred into sterile vials and frozen at −70°C ± 10°C. Analytical characterization included measurement of average particle diameter and degree of size heterogeneity of LNPs (ZEN3600, Malvern Instruments), RNA content, and encapsulation efficiency by a fluorometric assay using Ribogreen RNA reagent (Thermo Fisher Scientific).

### *Ex vivo* reactivation of HIV in primary CD4 T-cells by T66 mRNA

Two million CD4+ T-cells, isolated from PBMC of cART treated individuals, were stimulated for 48 hours with T66 mRNA packaged in LNP-2 (250 ng/mL, Arcturus) in presence of antiretroviral drugs (200 nM raltegravir, 200 nM lamivudine). A 24-hour stimulation with PMA (5 ng/mL) was included as positive control, while non-stimulated cells and cells incubated with HA LNP-2 (lipid nanoparticle formulation containing the hemagglutinin peptide from influenza) were used as negative controls. Following stimulation, cells were centrifuged (2,500 rpm, 10 min), and supernatant was collected for assessment of p24 release by p24-SIMOA (the protocol is described above). Cells were centrifuged at high speed (14,000 rpm, 10 min), and dry pellets were stored at –80°C until further processing. RNA extraction using the innuPREP RNA Mini Kit (Westburg, #AJ 845-KS-2040250) yielded a median concentration of 48.6 ng/µL (elution in a final volume of 30 µL). The reverse transcription (RT) and digital droplet PCR (ddPCR) steps were performed as described previously (99). In brief, the RT step was done in a final volume of 25 µL, and the mix was composed of: 2.5 µL 10× Superscript III buffer (Invitrogen #10308632), 2.5-µL MgCl2 50 mM, 1.25-µL random hexamers 50 ng/µL (Invitrogen #10308632), 1.25-µL dT15 50 µM, 1.25-µL dNTPs 10 mM, 0.625-µL RNAse OUT 40 U/µL (Invitrogen #10308632), 1.25-µL SuperScript III RT 200 U/µL (Invitrogen #10308632), and 14.4 µL of extracted RNA. Thermocycling conditions were as follows: 10 min at 25°C, 50 min at 50°C, and 5 min at 85°C. ddPCR on *tat/rev* transcripts was performed using the QX100 Droplet Digital qPCR System (Bio-Rad). The 20-µL PCR mix was composed of 10-µL ddPCR Probe Supermix (no dUTP), 1.8-µL *tat/rev* primers 900 nM (F: 5′-CTTAGGCATCTCCTATGGCAGGAA-3′, R: 5′-GGATCTGTCTCTGTCTCTCTCTCCACC-3′), 0.5-µL probe 250 nM (5′-ACCCGACAGGCC-3′), 0.9-µL H_2_O, and 5-µL undiluted RT product. Droplets were amplified (Thermal Cycler T100, Bio-Rad) using the following cycling conditions: 10 min at 95°C, 45 cycles (30 s at 95°C, and 60 s at 59°C), 10 min at 98°C. Each sample was measured in triplicate.

### Statistical analysis and software used

GraphPad Prism software (v9.0) was used to visualize data and perform statistical analysis. Kruskal-Wallis test is used to evaluate statistical significance within donors between different treatments. The Wilcoxon rank sum test was used to calculate the statistical significance of the relative gag RNA copy number between different conditions. For micro-array analysis, genes identified were considered statistically significant based on Benjamini-Hochberg adjusted *P*-value <0.05.

We used R software, version 3.6.1 (2019–07-05), platform x86_64-pc-linux-gnu (64-bit), running under Red Hat Enterprise Linux Server 7.9 (Maipo), and additionally utilized R packages, including Complex Heatmap (100) for data visualization.

## SUPPLEMENTAL MATERIAL

Supplemental figures aac.00417-23-s0001.docx Fig. S1 to S3

ASM does not own the copyrights to Supplemental Material that may be linked to, or accessed through, an article. The authors have granted ASM a non-exclusive, world-wide license to publish the Supplemental Material files. Please contact the corresponding author directly for reuse.
