# Supplementary material for: A truncated HIV Tat demonstrates potent and specific latency reversal activity
Source: Antimicrob Agents Chemother. 2023 Oct 24;67(11):e00417-23. doi: 10.1128/aac.00417-23 (PMC10649039; doi:10.1128/aac.00417-23)

**Supplementary Figures and legends**

**Fig. S1: influence of T66 protein on viability of primary CD4 T-cells.**

CD4+ T-cells from 6 HIV-seronegative donors were dosed with different concentrations of T66. Twenty-four hours later viability was determined.


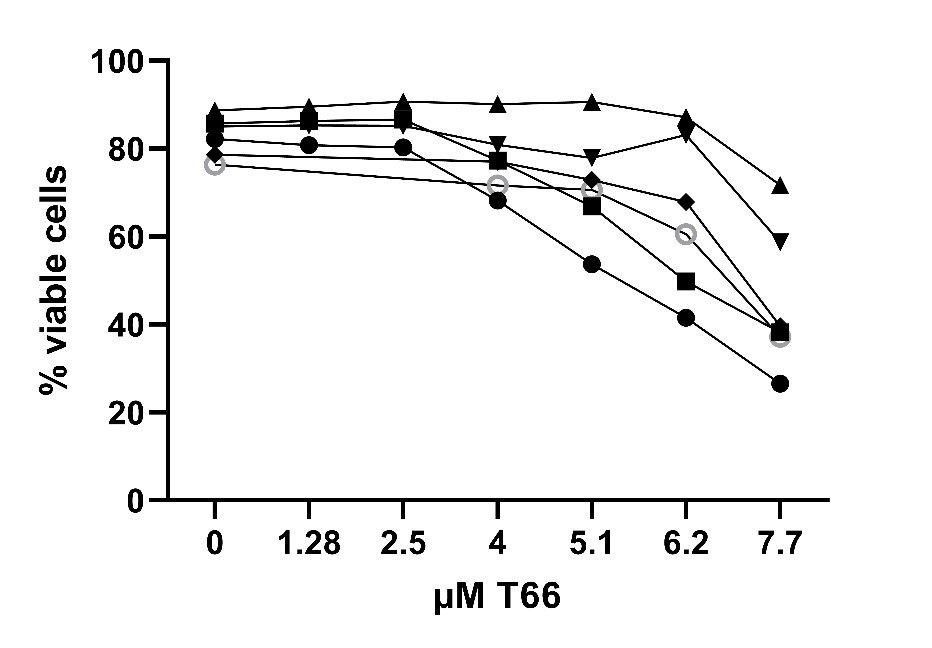


**Fig. S2: Positive controls used by the different labs showed equipotent HIV reactivation in CD4+ T-cells**

Primary CD4+ T-cells from 3 different HIV-seronegative donors (x-axis) were isolated and co-infected with an HIV dual reporter virus and a lentivirus expressing pro-survival proteins. Following infection, latently infected cells were sorted and exposed for 24h to various positive controls used by the different labs (black bar, PMA 5ng/ml; grey bar PHA 10µg/ml, patterned bar PHA 1.5µg/ml and light grey bar: anti-CD3/anti-CD28 beads~~)~~. On the Y-axis: increase in percentage of GFP positive cells compared to negative (PBS‑treated cells) is plotted. Statistical significance was evaluated by one-way ANOVA analysis.


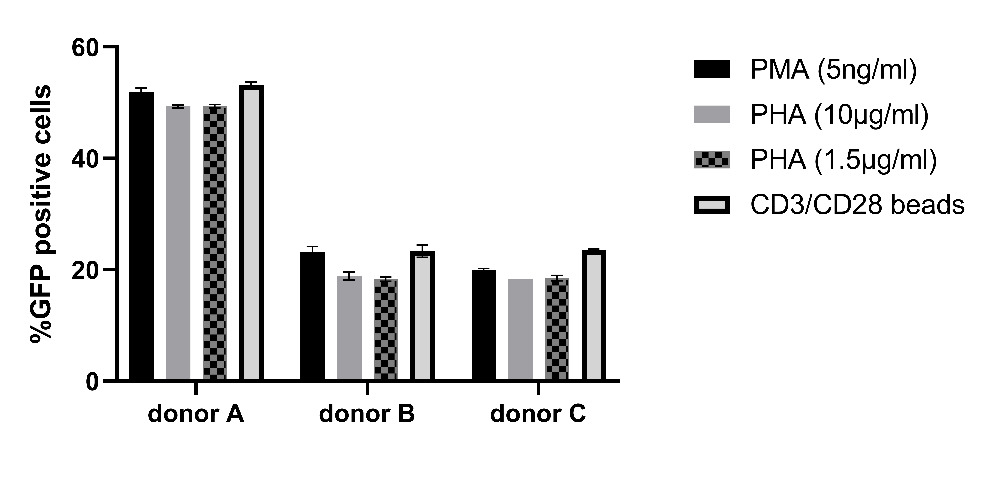


**Figure S3: T66 protein reactivates latently infected CD4+ T-cells as measured by** **cell-associated RNA** CD4+ T-cells from 4 cART treated individuals were exposed 24hours to PBS, T66 protein, or PHA. HIV-1 reactivation following latency reversal was assessed by RT-qPCR and presented here normalized to PHA activity. For each donor at least 12 experimental replicates were performed.


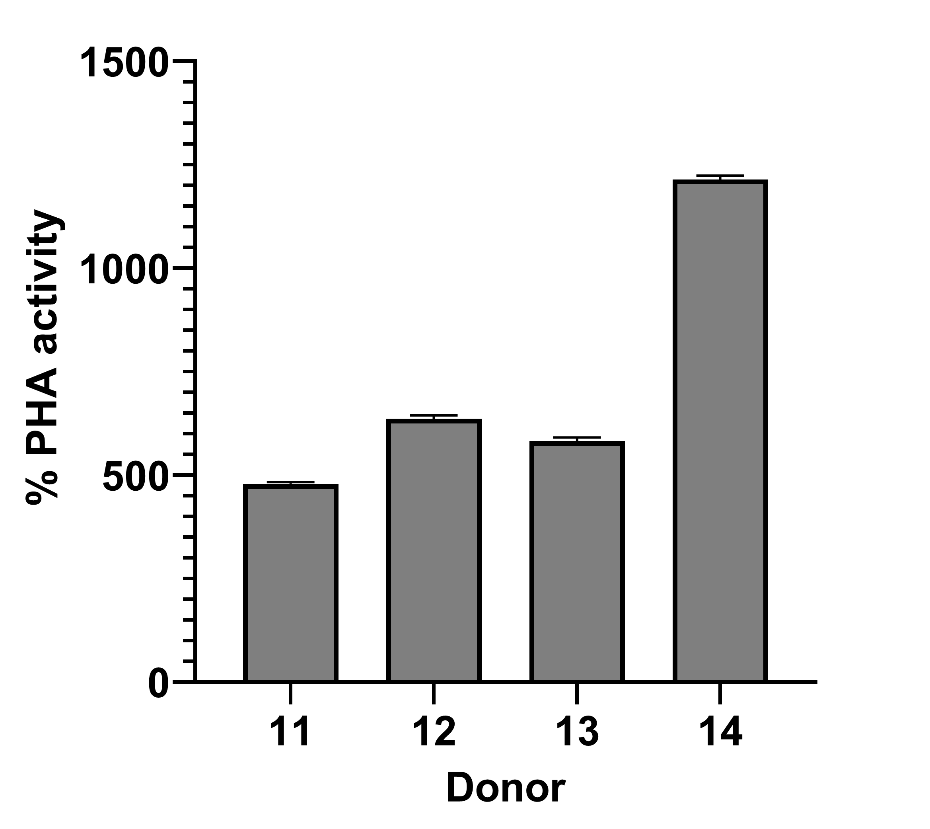

Supplement: Supplemental figures — Fig. S1 to S3 [file aac.00417-23-s0001.docx]
